# Comparative Physiological and Transcriptomic Analyses Reveal the Actions of Melatonin in the Delay of Postharvest Physiological Deterioration of Cassava

**DOI:** 10.3389/fpls.2016.00736

**Published:** 2016-05-27

**Authors:** Wei Hu, Hua Kong, Yunling Guo, Yuliang Zhang, Zehong Ding, Weiwei Tie, Yan Yan, Qixing Huang, Ming Peng, Haitao Shi, Anping Guo

**Affiliations:** ^1^Key Laboratory of Biology and Genetic Resources of Tropical Crops, Institute of Tropical Bioscience and Biotechnology, Chinese Academy of Tropical Agricultural SciencesHaikou, China; ^2^Hainan Key Laboratory for Sustainable Utilization of Tropical Bioresources, College of Agriculture, Hainan UniversityHaikou, China

**Keywords:** cassava, melatonin, postharvest physiological deterioration, reactive oxygen species, starch metabolism

## Abstract

Melatonin plays important roles in various aspects of biological processes. However, it is less known on the effects and mechanism of melatonin on the postharvest physiological deterioration (PPD) process of cassava, which largely restricts the potential of cassava as a food and industrial crop. In this study, we found that exogenous application of melatonin significantly delayed PPD of cassava tuberous roots by reducing H_2_O_2_ content and improving activities of catalase and peroxidase. Moreover, 3425 differentially expressed genes by melatonin during the PPD process were identified by transcriptomic analysis. Several pathways were markedly affected by melatonin treatments, including metabolic-, ion homeostasis-, and enzyme activity-related processes. Further detailed analysis revealed that melatonin acted through activation of ROS-scavenging and ROS signal transduction pathways, including antioxidant enzymes, calcium signaling, MAPK cascades, and transcription factors at early stages. Notably, the starch degradation pathway was also activated at early stages, whereas it was repressed by melatonin at middle and late stages, thereby indicating its regulatory role in starch metabolism during PPD. Taken together, this study yields new insights into the effect and underlying mechanism of melatonin on the delay of PPD and provides a good strategy for extending shelf life and improvement of cassava tuberous roots.

## Introduction

In the late 1950s, melatonin was first identified from the borine pineal gland (Lerner et al., 1958, 1959). In animals, melatonin exerts various biological roles, including effects on sleep physiology, circadian rhythms, sexual behavior, mood, immune system, body temperature regulation, and seasonal reproduction (Cardinali et al., 2012; Calvo et al., 2013; Reiter et al., 2014). In addition, melatonin was reported to be a direct scavenger of reactive oxygen species (ROS) and of other free radicals, but also through activating antioxidant enzymes and augmenting the efficiency of other antioxidants (Tan et al., 1993, 2003; Barlow-Walden et al., 1995; Pablos et al., 1998; Rodriguez et al., 2004; Hardeland, 2005; Kolář and Macháčkova, 2005; Reiter et al., 2009; Galano et al., 2013; Zhang and Zhang, 2014). Dubbels et al. (1995), and Hattori et al. (1995) melatonin was discovered in plants. Further, endogenous concentrations of melatonin were found to be different in plants as function of growth state, growth location, plant tissue, and harvest time (Hattori et al., 1995; Burkhardt et al., 2001; Pape and Lüning, 2006; Hernández-Ruiz and Arnao, 2008; Arnao and Hernández-Ruiz, 2009, 2013; Murch et al., 2009; Okazaki et al., 2010; Ramakrishna et al., 2012; Wang L. et al., 2014). Subsequently, increasing evidences demonstrated that melatonin is involved in multiple biological processes in plants, such as seed germination, photo-protection, vegetative growth, flower development, leaf senescence, root architecture, fruit ripening, and response to biotic and abiotic stresses (Manchester et al., 2000; Arnao and Hernández-Ruiz, 2006; Paredes et al., 2009; Tan et al., 2012; Wang et al., 2012, 2013a,b; Zhao et al., 2013; Shi and Chan, 2014; Wang P. et al., 2014; Zhang et al., 2014b; Shi et al., 2015a,b). Because melatonin can act as an important antioxidant in animals, many studies emphasized the importance of melatonin in directly or indirectly scavenging ROS in plants (Arnao et al., 2001; Cano et al., 2006).

Cassava is the third most important crop besides rice and maize in Africa, Asia, and Latin America (Oliveira et al., 2014; Hu et al., 2015a,b). Due to its high starch content and limited input needs, cassava can provide a source of dietary carbohydrate for over 600 million people worldwide, and is also considered as a producer of industrial starch and bioethanol (Zidenga et al., 2012). The potential of cassava as a food and industrial crop is largely restricted by the rapid postharvest physiological deterioration (PPD) of the tuberous root that starts within 72 h after harvest (Zidenga et al., 2012; Vanderschuren et al., 2014). PPD is induced by mechanical injury, handling operations, and storage conditions (Vanderschuren et al., 2014). To this end, more attention has been paid to the physiology and biochemistry of PPD (Reilly et al., 2001, 2004; Iyer et al., 2010; Zidenga et al., 2012; Xu et al., 2013; Vanderschuren et al., 2014). These studies have revealed that ROS production is one of the earliest events in the PPD process, and reduction of ROS accumulation by overexpression of scavenging enzymes could lead to the delay of PPD. However, less is known on the effect and mechanism of melatonin on the PPD process of cassava tuberous roots.

In this study, to gain insight into the roles and regulatory mechanism of melatonin in PPD of cassava, we investigated the effect of melatonin on the phenotype, physiology, and gene transcription rate during the postharvest period of cassava tuberous roots, and found that melatonin plays a role in the delay of PPD by reducing ROS and improving ROS signaling network. We also found that exogenous application of melatonin alters the expression of genes involved in the starch metabolism pathway during the PPD process. This study provides insights into the roles and molecular mechanisms of the actions of melatonin in the delay of PPD in cassava.

## Materials and Methods

### Plant Material and Treatment

Cassava tuberous roots were harvested from 10-month-old cassava varieties SC124 (*Manihot esculenta* cv SC124) grown in a greenhouse (60% humidity, 16 h light, 35/20°C). The tuberous roots were separated into two sample groups, water-treated PPD and melatonin-treated PPD. For water-treated PPD, whole tuberous roots were soaked in water for 2 h; for melatonin-treated PPD, whole tuberous roots were soaked in 100 μM melatonin for 2 h. Tuberous roots were cut into slices approximately 5 mm thick, then were moved into petri dishes containing a wet filter paper, and then were incubated at 28°C and 60% relative humidity in the dark (Vanderschuren et al., 2014). After incubation for 0, 6, 12, 24, 48, and 72 h, the slices were sampled and frozen in liquid nitrogen until extraction of total RNA and transcriptomic analysis. Each sample contains four replicates from independent experiments.

### Determination of H_2_O_2_ Content and Antioxidant Enzyme Activities

The activities of peroxidase (POD), catalase (CAT), and superoxide dismutase (SOD) and H_2_O_2_ content were measured spectrophotometrically. Approximately 0.5 g of roots slices was ground and homogenized in 5 mL of extraction buffer with 0.05 M phosphate buffer (pH7.8). The homogenate was centrifuged at 10000 *g* for 10 min at 4°C and the resulting supernatant was collected for analysis of H_2_O_2_ content and enzyme activities. Total POD activity was examined according to the changes in absorbance at 470 nm (Polle et al., 1994). H_2_O_2_ content and activities of CAT and SOD were detected using H_2_O_2_, CAT and SOD Detection Kits (A064, A007 and A001, Nanjing Jiancheng, Nanjing city, China) according to manufacturer’s instructions. Each sample contains four replicates consisting of three root slices each.

### Transcriptomic Analysis

Total RNA was extracted from tuberous roots of SC124 variety after 6, 12, and 48 h of incubation with or without melatonin according to the manufacturer’s instructions for its plant RNA extraction kit (DP432, TIANGEN, Beijing city, China). Each sample contains three replicates from independent experiments. Three micrograms of total RNA from each sample were converted into cDNA with an AMV Reverse Transcriptase (Promega, Madison, WI, USA). Eighteen cDNA libraries were generated and sequenced by the use of Illumina GAII following the Illumina RNA-seq protocol. A total of 1667.1 million 51-bp raw reads was produced from the 18 samples. Using FASTX-toolkit, adapter sequences in the raw sequence reads were removed. After examining the sequence quality and removing low quality sequences by FastQC, 1589.5 million clean reads were generated. Using Tophat v.2.0.10, 84.2% clean reads were mapped to the cassava reference genome (version 4.1) (Trapnell et al., 2009). The transcriptome assemblies were performed by Cufflinks with alignment files (Trapnell et al., 2012). Gene expression levels were calculated as Fragments Per Kilobase of transcript per Million mapped reads (FPKM). Differentially expressed genes (DEGs) were identified by DEGseq according to the read count for each gene (Wang et al., 2010).

### Statistical Analysis

Statistical analyses were carried out with SPSS (SPSS Inc., Chicago, IL, USA). Physiological data was analyzed by Duncan’s multiple range test. Means denoted by the same letter do not significantly differ at *P* < 0.05 (lower case letters) or *P* < 0.01 (upper case letters) (*n* = 4). Transcriptomic data was analyzed using Student’s *t*-tests at a significance level of ^∗^*P* < 0.05, ^∗∗^*P* < 0.01, and ^∗∗∗^*P* < 0.001 (*n* = 3).

## Results

### Exogenous Application of Melatonin Delayed PPD of Cassava Tuberous Roots

**FIGURE 1:**
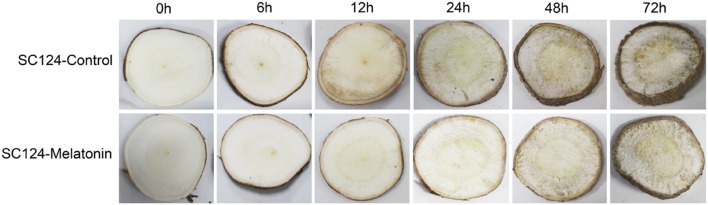
**Effect of melatonin on PPD of tuberous roots in SC124 variety.** After soaking water (control) or 100 μM melatonin (melatonin treatment) for 2 h, cassava tuberous roots were sliced into 5-mm-thick slices, following incubation in constant temperature of 28°C in the dark. Photos were taken at 0, 6, 12, 24, 48, and 72 h after treatment.

To investigate the effect of melatonin on PPD of cassava tuberous roots, SC124 variety widely planted in China were treated with water (control) or melatonin for 2 h, then the PPD symptoms were observed at 0, 6, 12, 24, 48, and 72 h after treatment (**Figure 1**). After 6 h of water treatment, PPD symptoms could be observed in the tuberous roots with slight ‘vascular streaking.’ Further, ‘vascular discoloration’ symptom with brown color becoming spread on the whole surface of cassava tuber slices became visible after 12 h of water treatment. In comparison to water treatment, melatonin-treated cassava tuberous roots showed ‘vascular streaking’ after 12 h of treatment and ‘vascular discoloration’ after 48 h of treatment. These results indicate that exogenous application of melatonin delays PPD occurrence in cassava tuberous roots.

### Exogenous Application of Melatonin Decreased H_2_O_2_ Content and Improved Activities of CAT and POD during the PPD Process

**FIGURE 2:**
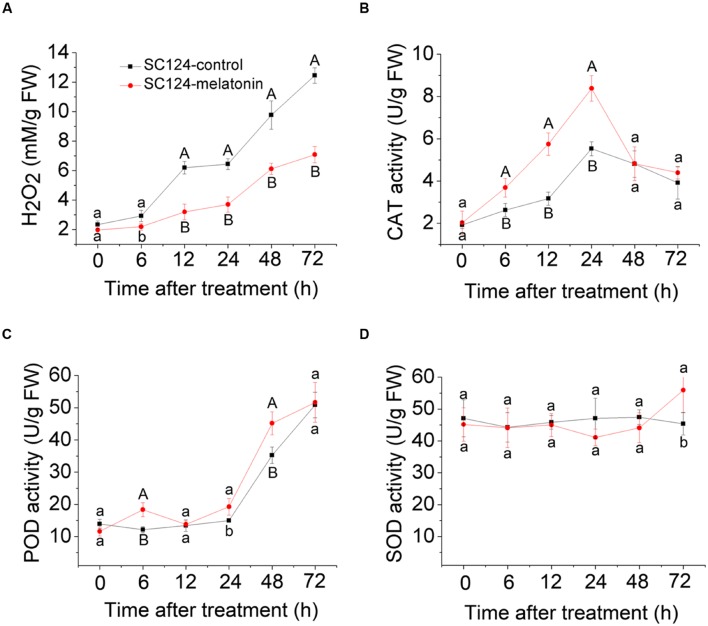
**Effect of melatonin on H_2_O_2_ accumulation **(A)** and activities of CAT **(B)**, POD **(C)**, and SOD **(D)** during the PPD process in SC124 variety.** Data are means ± SE calculated from four biological replicates. Means denoted by the same letter do not significantly differ at *P* < 0.05 (lower case letters) or *P* < 0.01 (upper case letters) as determined by Duncan’s multiple range test.

To test whether melatonin-induced delay in PPD is related to ROS scavenging in tuberous roots during the postharvest period, H_2_O_2_ content was examined at different time points. Generally, H_2_O_2_ content increased in the tuberous roots of SC124 during 0–72 h postharvest with or without melatonin treatment, indicating the increasing of oxidative damage during postharvest. Compared with water-treated samples, melatonin-treated cassava tuberous roots showed lower H_2_O_2_ accumulation during the 6 to72 h postharvest period (**Figure 2**).

To detect whether melatonin-induced ROS scavenging is related to the activities of antioxidative enzymes during the postharvest period, CAT, POD, and SOD activities were tested at different time points (**Figure 2**). During the PPD process, CAT activity was significantly higher in melatonin-treated cassava tuberous roots than that in control samples at stages of 6, 12, and 24 h after treatment. Additionally, POD activity significantly increased in melatonin-treated samples compared with controls at stages of 6, 24, and 48 h after treatment. There were no significant differences for SOD activity between controls and melatonin-treated samples. These results indicate that increased activities of CAT and POD may be involved in melatonin-induced ROS scavenging during the PPD process.

### Differential Expression Profiling between Water-Treated and Melatonin-Treated Cassava Tuberous Roots

**FIGURE 3:**
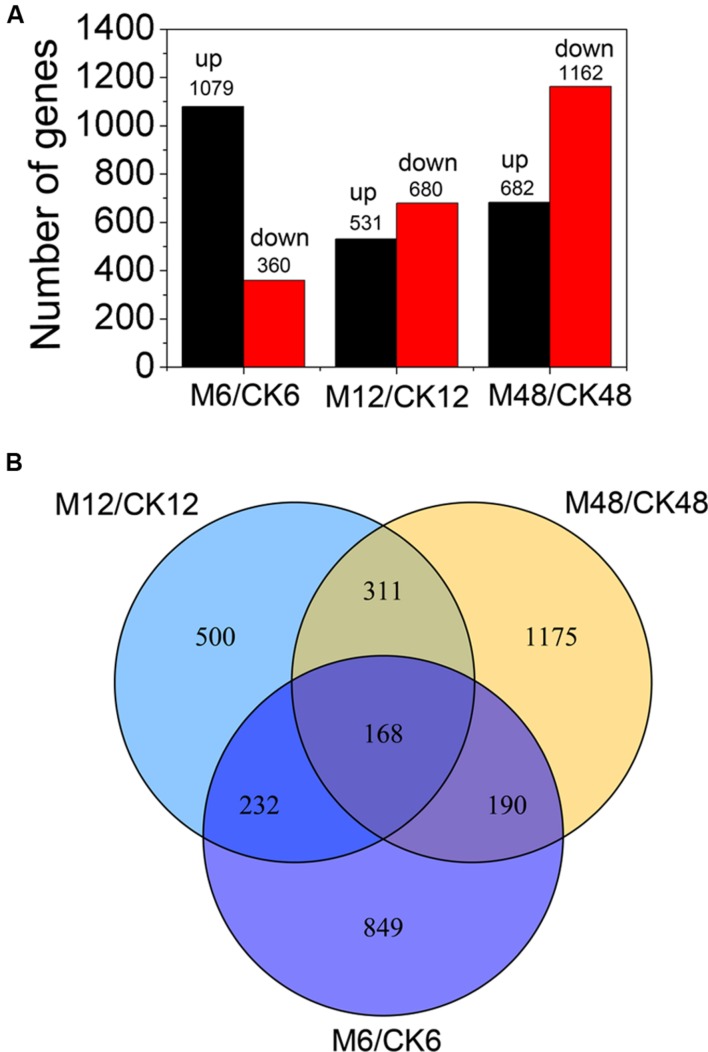
**Distribution of differentially expressed genes at different storage stages after melatonin treatment.** **(A)** The number of differentially expressed genes (fold change ≥ 2; *P*-value ≤ 0.05). **(B)** Differentially expressed genes overlapped at different storage stages after melatonin treatment by venn diagram analysis. M6/CK6, melatonin-6 h/control check-6 h; M12/CK12, melatonin-12 h/control check-12 h; M48/CK48, melatonin-48 h/control check-48 h.

To study the transcriptionally regulatory mechanism underlying the melatonin-mediated delay of PPD in cassava, we performed comparative transcriptomic analysis between water-treated and melatonin-treated SC124 cassava tuberous roots during PPD. A total of 3425 differentially expressed genes (DEGs) (fold change ≥2; *P*-value ≤0.05) were identified by exogenous melatonin treatment, including 1439 (1079 up- and 360 down-regulated) genes from M6/CK6, 1211 genes from M12/CK12 (531up- and 680 down-regulated), and 1844 genes from M48/CK48 (682 up- and 1162 down-regulated) (**Figure 3A**; **Supplementary Tables S1**–**S4**). Gene ontology (GO) enrichment analysis showed that many metabolic-, ion homeostasis-, and enzyme activity-related pathways were extensively changed after melatonin treatment during PPD (**Supplementary Figure S1**; **Supplementary Table S5**). Notably, some genes associated with response to oxidative stress, catalytic activity, antioxidant activity, and oxidation-reduction were enriched, indicating that melatonin-induced antioxidant response was activated during PPD process.

Additionally, venn diagram analysis showed the amounts of 168 DEGs (fold change ≥ 2; *P*-value ≤ 0.05) present in all three stages, indicating that these genes were involved in the melatonin-mediated delay of PPD during 6–48 h (**Figure 3B**). Among these, some genes related to ROS scavenging were found, suggesting their crucial roles in the melatonin-mediated delay of PPD in cassava tuberous roots (**Supplementary Table S6**). GO enrichment analysis indicated that several pathways were significantly affected by melatonin treatments, including carbohydrate metabolic process, polysaccharide catabolic/metabolic process, biological process, glucosidase activity, hydrolase activity, and catabolic activity (Conesa et al., 2005) (**Supplementary Figure S2**; **Supplementary Table S7**).

### Melatonin-Mediated ROS Scavenging Activity by Antioxidant Enzymes during PPD Process

**FIGURE 4:**
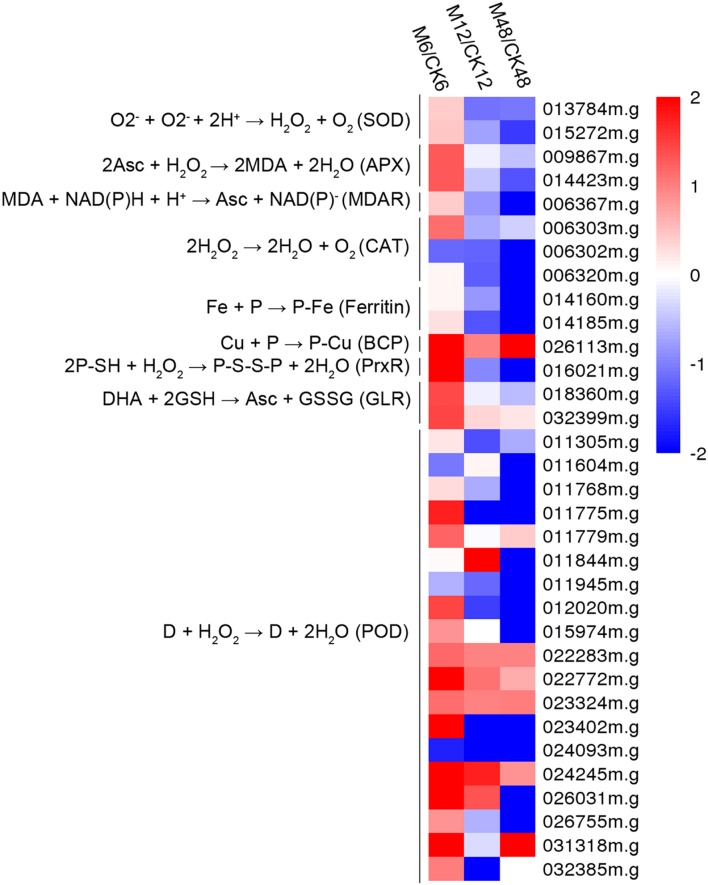
**Effects of melatonin on the expression of genes encoding antioxidant enzymes.** Thirty-three genes encoding antioxidant enzymes were identified from the DEGs (fold change ≥2; *P*-value ≤0.05 at least in one stage of PPD). Log2 based Fragments Per Kilobase of transcript per Million mapped reads (FPKM) value was used to create the heat map. The scale represents the relative signal intensity of FPKM values. M6/CK6, melatonin-6 h/control check-6 h; M12/CK12, melatonin-12 h/control check-12 h; M48/CK48, melatonin-48 h/control check-48 h; SOD, superoxide dismutase; APX, ascorbate peroxidase; MDAR, monodehydroascorbate reductase; CAT, catalase; BCP, blue copper protein; PrxR, peroxiredoxin; GLR, glutaredoxin; POD, peroxidase.

To better understand the action of melatonin on ROS scavenging during the PPD process, 34 genes encoding antioxidant enzymes were identified from the DEGs (fold change ≥2; *P*-value ≤0.05 at least in one stage of PPD). Heat map analysis indicated that 30, 10, and 8 genes were up-regulated after 6, 12, and 48 h melatonin treatment, respectively; whereas 4, 24, and 25 genes were down-regulated after 6, 12, and 48 h of melatonin treatment, respectively (**Figure 4**; **Supplementary Table S8**). From these data, it can be concluded that many genes were transcriptionally induced at an early storage stage (6 h), whereas they were repressed following middle (12 h) and late stages (48 h), indicating the rapid activation the expression of genes encoding antioxidant enzymes by melatonin.

### Melatonin-Mediated ROS Signaling Network during the PPD Process

**FIGURE 5:**
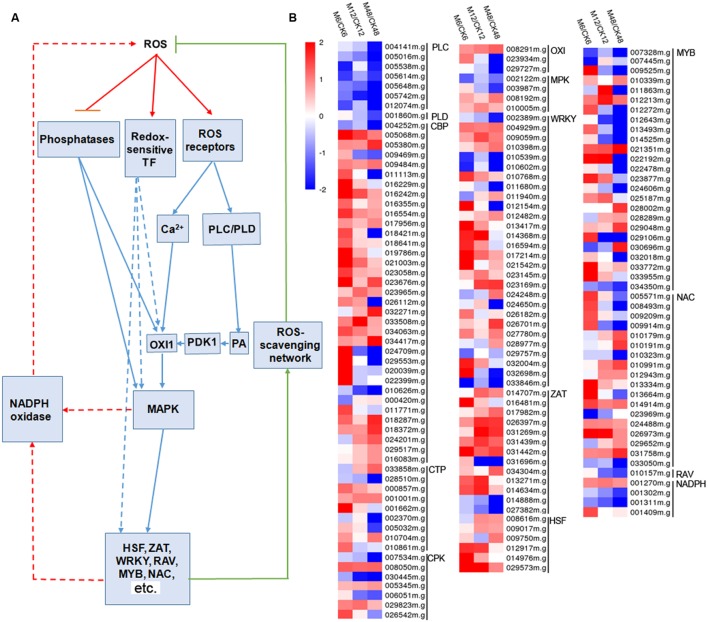
**Effects of melatonin on the expression of genes involved in the ROS signaling network.** **(A)** Generalized model of the ROS signal transduction pathway. **(B)** Expression patterns of the genes involved in the ROS signaling network after melatonin treatment. 161 genes associated with ROS signaling network were identified from the DEGs (fold change ≥2; *P*-value ≤0.05 at least in one stage of PPD). Log2 based FPKM value was used to create the heat map. The scale represents the relative signal intensity of FPKM values. M6/CK6, melatonin-6 h/control check-6 h; M12/CK12, melatonin-12 h/control check-12 h; M48/CK48, melatonin-48 h/control check-48 h; PLC, phospholipase C; PLD, phospholipase D; CBP, calcium-binding protein; CTP, calcium-transporting protein; CPK, calcium-related protein kinase; OXI, oxidative signal-inducible protein; PDK1, PDK, phosphoinositide-dependent kinase; PA, phosphatidic acid; MPK, mitogen-activated protein kinase cascades; WRKY, WRKY transcription factor; ZAT, zinc finger protein; HSF, heat stress transcription factor; MYB, MYB transcription factor; NAC, NAC transcription factor; AP2/ERF and B3 domain-containing transcription factor; NADPH, NADPH oxidase.

To gain insight into the melatonin-mediated ROS signaling network during the PPD process, 161 genes related to ROS response were identified from the DEGs (fold change ≥2; *P*-value ≤0.05 at least in one stage of PPD), including calcium signaling-, phospholipase signaling-, MAPK cascades-, NADPH oxidase-, and transcription factor-related genes (**Figure 5**; **Supplementary Table S9**). Expression profiles showed that 114, 98, and 83 genes showed up-regulation after 6, 12, and 48 h melatonin treatment, respectively; whereas 47, 61, and 76 genes showed down-regulation after 6, 12, and 48 h melatonin treatment, respectively. This indicates that more ROS-responsive genes were activated at early stages relative to middle and late stages by melatonin.

### Melatonin-Mediated Starch Metabolism during the PPD Process

**FIGURE 6:**
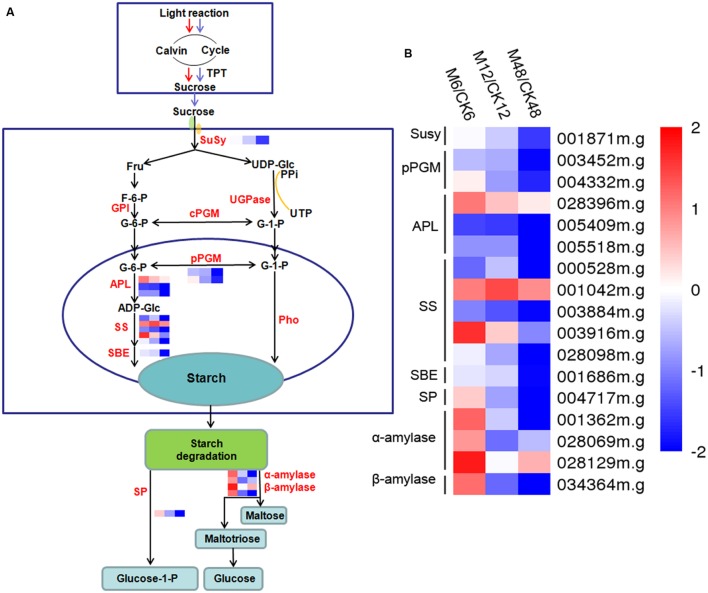
**Effects of melatonin on the expression of genes associated with the starch metabolism pathway.** **(A)** Generalized model of starch metabolism pathway in cassava tuberous roots. **(B)** Expression patterns of the genes associated with starch metabolism pathway after melatonin treatment. Seventeen genes related to starch metabolism were identified from the DEGs (fold change ≥2; *P*-value ≤0.05 at least in one stage of PPD). Log2 based FPKM value was used to create the heat map. The scale represents the relative signal intensity of FPKM values. M6/CK6, melatonin-6 h/control check-6 h; M12/CK12, melatonin-12 h/control check-12 h; M48/CK48, melatonin-48 h/control check-48 h; Susy, sucrose synthase; pPGM, glucose phosphomutase; APL, ADP-glucose pyrophosphorylase; SS, starch synthase; SBE, starch branching enzyme; SP, starch phosphorylase.

To get some clues on the roles of melatonin in regulating the starch synthesis and degradation processes, 17 starch metabolic associated genes were identified from the DEGs (fold change ≥ 2; *P*-value ≤ 0.05 at least in one stage of PPD). Transcriptomic data indicated that 9, 3, and 3 genes were up-regulated after 6, 12, and 48 h melatonin treatment, respectively; whereas 8, 14, and 14 genes were down-regulated after 6, 12, and 48 h melatonin treatment, respectively (**Figure 6**; **Supplementary Table S10**). Genes related to starch synthesis, including sucrose synthase, glucose phosphomutase, and ADP-glucose pyrophosphorylase were mainly repressed, indicating the repression of starch synthesis process by melatonin during PPD. Most of genes involved in starch degradation, such as starch phosphorylase, alpha-amylase, and beta-amylase were induced at the early stage (6 h); however, repressed at the middle and late stages (12 and 48 h). These results suggest that melatonin may be a crucial regulator of starch metabolism during PPD process of cassava tuberous roots.

## Discussion

Cassava is one of the most important crop in Africa, Asia, and Latin America. Nevertheless, the rapid PPD of its tuberous roots, a unique phenomenon compared with other root crops, renders the roots unpalatable and unmarketable, thereby adversely impacting farmers, processors, and consumers alike (Xu et al., 2013). Previous studies demonstrated that ROS production is one of the earliest events in the deterioration process and reduction of ROS accumulation could lead to a delayed onset of PPD (Reilly et al., 2001, 2004; Iyer et al., 2010; Zidenga et al., 2012; Xu et al., 2013; Vanderschuren et al., 2014). Melatonin was reported to function in the reduction of ROS through scavenging ROS directly and enhancing the levels of antioxidants and the activities of antioxidative enzymes (Tan et al., 1993, 2003; Barlow-Walden et al., 1995; Pablos et al., 1998; Rodriguez et al., 2004; Hardeland, 2005; Kolář and Macháčkova, 2005; Reiter et al., 2009; Galano et al., 2013; Zhang and Zhang, 2014; Zhang et al., 2014a; Shi et al., 2015d). Therefore, we hypothesized that exogenous application of melatonin might delay PPD of cassava tuberous roots by scavenging ROS. To confirm this, cassava tuberous roots were treated with melatonin. Compared with controls, melatonin-treated cassava tuberous roots showed delayed PPD both for ‘vascular streaking’ and ‘vascular discoloration’ symptoms, indicating that melatonin has a clear effect on PPD (**Figure 1**). Physiological analyses also support that melatonin-mediated delay of PPD is related to reduced H_2_O_2_ and increased activities of CAT and POD (**Figure 2**).

To provide the possible molecular evidence, transcriptomic analysis was carried out between water-treated and melatonin-treated cassava tuberous roots during the PPD process. Among the DEGs, 30 out of 34 genes encoding enzymes associated with ROS scavenging, including *SOD*, *POD*, *CAT*, *APX*, *PrxR*, were transcriptionally induced by melatonin at early storage stage (6 h) during PPD (**Figure 4**; **Supplementary Table S8**). These genes provide cells with highly efficient machinery for detoxifying O2^-^ and H_2_O_2_ in plants (Mittler et al., 2004). Accordingly, the decreased H_2_O_2_ content, increased activities of POD and CAT, and lightened PPD symptoms after melatonin treatment were also observed at early storage stage (6 h) during PPD (**Figures 1** and **2**). These results further support that the melatonin mediated ROS-scavenging system may contribute to reducing oxidative injury of cassava tuberous roots.

Previous studies demonstrated that an oxidative burst occurred at the early stage of PPD (Reilly et al., 2001, 2004; Iyer et al., 2010; Zidenga et al., 2012; Xu et al., 2013; Vanderschuren et al., 2014). Coincidently, melatonin acted through scavenging ROS by antioxidant enzymes, thus assisting in reduction of cellular ROS levels at early stage. After oxidative burst, cells still need to maintain a homeostasis of ROS. Thus, the downregulation of genes encoding ROS-scavenging enzymes at middle (12 h) and late stages (48 h) by melatonin implied that melatonin may be a flexible regulator of ROS during the PPD process (**Figure 4**; **Supplementary Table S8**). In addition, although melatonin repressed the expression of genes encoding ROS-scavenging enzymes at middle and late stages, melatonin-treated samples still maintained lower H_2_O_2_ levels and higher activities of CAT and POD relative to control samples (**Figures 1** and **2**). This implies that melatonin may extend the effects of early transcriptional activation to enzyme activity levels at middle and late stages through a series of post-transcriptionally regulatory mechanisms.

Plant cells sense ROS through at least three different mechanisms, including unidentified receptor proteins, redox-sensitive transcription factors, and direct inhibition of phosphatases. Downstream signaling events include calcium and phospholipid signaling pathways, and hence activate oxidative signal-inducible protein (OXI1), MAPK cascades, NADPH oxidase, and transcription factors (Mittler et al., 2004; Kim et al., 2011). To address the question how does melatonin regulate cells to perceive and transduct ROS signaling, those genes related to the ROS network were identified in the melatonin-treated cassava tuberous roots. Notably, many calcium signaling pathway connected genes, including calcium-sensing, -transport, -transduction genes, and downstream transcription factors, were significantly induced, whereas almost all of the phospholipase C (*PLC*) and phospholipase D (*PLD*) genes in phospholipid signaling pathway were repressed by melatonin during the PPD process (**Figure 5**; **Supplementary Table S9**). Accordingly, phosphoinositide-dependent kinase (PDK1), a downstream component of phospholipid signaling pathway, did not show significant difference at transcriptional levels with melatonin treatment. This suggests that melatonin-induced ROS signaling is positively transducted through calcium signaling components. In response to melatonin, 14 genes related to calcium-dependent signaling were induced in *Arabidopsis* by RNA-seq analysis (Weeda et al., 2014). Calcium-related protein kinase (CRK), calcium-dependent protein kinase (CDPK), and calcineurin B-like (CBL)-interacting protein kinase (CIPK) were reported to be commonly regulated by melatonin treatment in Bermuda grass (Shi et al., 2015a). Many transcription factors, including WRKY, NAC, ZAT, and HSF were up-regulated after melatonin treatment and some of them were confirmed to play a melatonin-mediated protective role in abiotic stress response and leaf senescence by functional analyses (Shi and Chan, 2014; Zhang et al., 2014b; Shi et al., 2015a,c). Also, the crosstalk between melatonin and calcium signaling has been demonstrated to modulate various calcium-dependent cellular functions in animal cells (Posmyk and Janas, 2009). Additionally, calmodulin proteins were also reported to be up-regulated during early PPD of cassava (Owiti et al., 2011). Therefore, the extensive activation of calcium signaling and related transcription factors may be an important event in melatonin-mediated ROS pathway during PPD.

Besides, we also noted that the number of genes related to ROS network induced by melatonin was greater at early stages than that at middle and late stages (**Figure 5**; **Supplementary Table S9**). This is consistent with the expression patterns of genes encoding ROS-scavenging enzymes after melatonin treatment in general (**Figure 4**; **Supplementary Table S8**). Based on this consistent trend, it is concluded that coincidently with an oxidative burst melatonin acted by inducing cells to sense and transduct ROS signaling through calcium signaling and related transcription factors, thus resulting in the induction of ROS-scavenging genes to relieve ROS injury.

Interestingly, we observed that numerous starch metabolism-related genes showed transcriptional changes after melatonin treatment according to GO analyses (**Supplementary Figures S1** and **S2**). Most of genes related to starch synthesis were repressed during the PPD process (**Figure 6**; **Supplementary Table S10**). After harvest, the primary carbon source supplied by photosynthesis was cut off, which limited the function of starch synthesis-related genes. The repression of starch synthesis pathway by melatonin may represent an action of melatonin-mediated saving of cell resources in tuberous roots after harvest.

Additionally, exogenous application of melatonin resulted in upregulation of starch degradation related genes at early stage of PPD (6 h) (**Figure 6**; **Supplementary Table S10**), which is consistent with the expression profiles of genes in ROS-scavenging and ROS signal transduction pathways. Starch degradation produces glucose that supplies energy for various biological process. It is possible that melatonin-mediated starch degradation may provide energy for the active ROS-scavenging process, such as ascorbate-glutathione cycle, at early stage of PPD.

As reported by Uarrota et al. (2016), degradation of starch occurred during the PPD process, which resulted in reducing starch content, thus decreasing the commercial value of cassava. Exogenous application of melatonin decreased the transcripts of starch degradation associated genes at the middle and late stages (12 and 48 h) of postharvest (**Figure 6**; **Supplementary Table S10**). This indicates that melatonin cannot only alleviate PPD symptoms, but also can synchronously inhibit starch degradation, which is of great importance for improving food characteristics and quality of cassava. ROS have been implicated in the oxidative-reductive depolymerization of carbohydrates (Uarrota et al., 2016). The early activation of ROS-scavenging events by melatonin may contribute to the subsequent repression of starch degradation during PPD process.

Recent studies suggested that low doses of melatonin accelerated starch catabolism at night, whereas high doses of melatonin significantly decreased this process and led to starch accumulation in photosynthetic tissues of maize (Zhao et al., 2015). Additionally, melatonin could alter the metabolic status and increase the levels of starch in leaf tissue of *Malus hupehensis* (Wang et al., 2013a). These studies indicate the physiolgical effect of melatonin on starch metabolism in photosynthetic tissues. The evidences presented here expand the role of and transcriptionally regulatory mechanism of melatonin on starch metabolism in non-photosynthetic tissue.

## Conclusion

**FIGURE 7:**
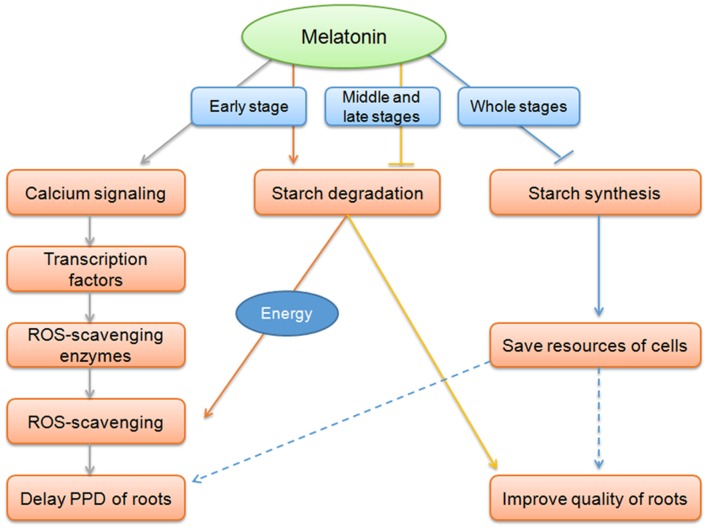
**Overview of melatonin action on the delay of PPD of cassava tuberous roots.** At early stage of the PPD process, coincidently with oxidative burst, melatonin acted on inducing cells to sense and transduct ROS signaling through calcium signaling and related transcription factors, thus resulting in the induction of ROS-scavenging genes to relieve ROS injury and delay of PPD. Melatonin-mediated starch degradation may provide energy for the active ROS-scavenging process at early stage of PPD. At middle and late stages, melatonin function on repressing starch degradation, benefit for improving food characteristics and quality of cassava. During all three stages, the repression of starch synthesis pathway by melatonin may represent an action of melatonin-mediated saving resources of cells in tuberous roots.

This study demonstrated the effect of melatonin on the delay of PPD in cassava tuberous roots is related to ROS-scavenging pathway, ROS-signaling transduction, and starch metabolism (**Figure 7**). These findings would contribute to extend shelf life and improve quality of casssava tuberous roots.

## Author Contributions

HS, HK, MP, and AG conceived the study. WH, YG, YZ, and YY performed the experiments. ZD, QH, and WT carried out the analysis. WH and HS designed the experiments and wrote the manuscript.

## Conflict of Interest Statement

The authors declare that the research was conducted in the absence of any commercial or financial relationships that could be construed as a potential conflict of interest.

## Supplementary Material

**FIGURE S1 | Differentially expressed genes at 6, 12, and 48 h storage after melatonin treatment by GO enrichment analysis.** Asterisks indicate a significant difference (^∗^*P* < 0.05; ^∗∗^*P* < 0.01; ^∗∗∗^*P* < 0.001).

**FIGURE S2 | Common genes differentially expressed at 6, 12, and 48 h storage after melatonin treatment by GO enrichment analysis.** Asterisks indicate a significant difference (^∗^*P* < 0.05; ^∗∗^*P* < 0.01; ^∗∗∗^*P* < 0.001).
