# Supplementary figures and images for: Comparative Physiological and Transcriptomic Analyses Reveal the Actions of Melatonin in the Delay of Postharvest Physiological Deterioration of Cassava
Source: Front Plant Sci. 2016 May 27;7:736. doi: 10.3389/fpls.2016.00736 (PMC4882330; doi:10.3389/fpls.2016.00736)

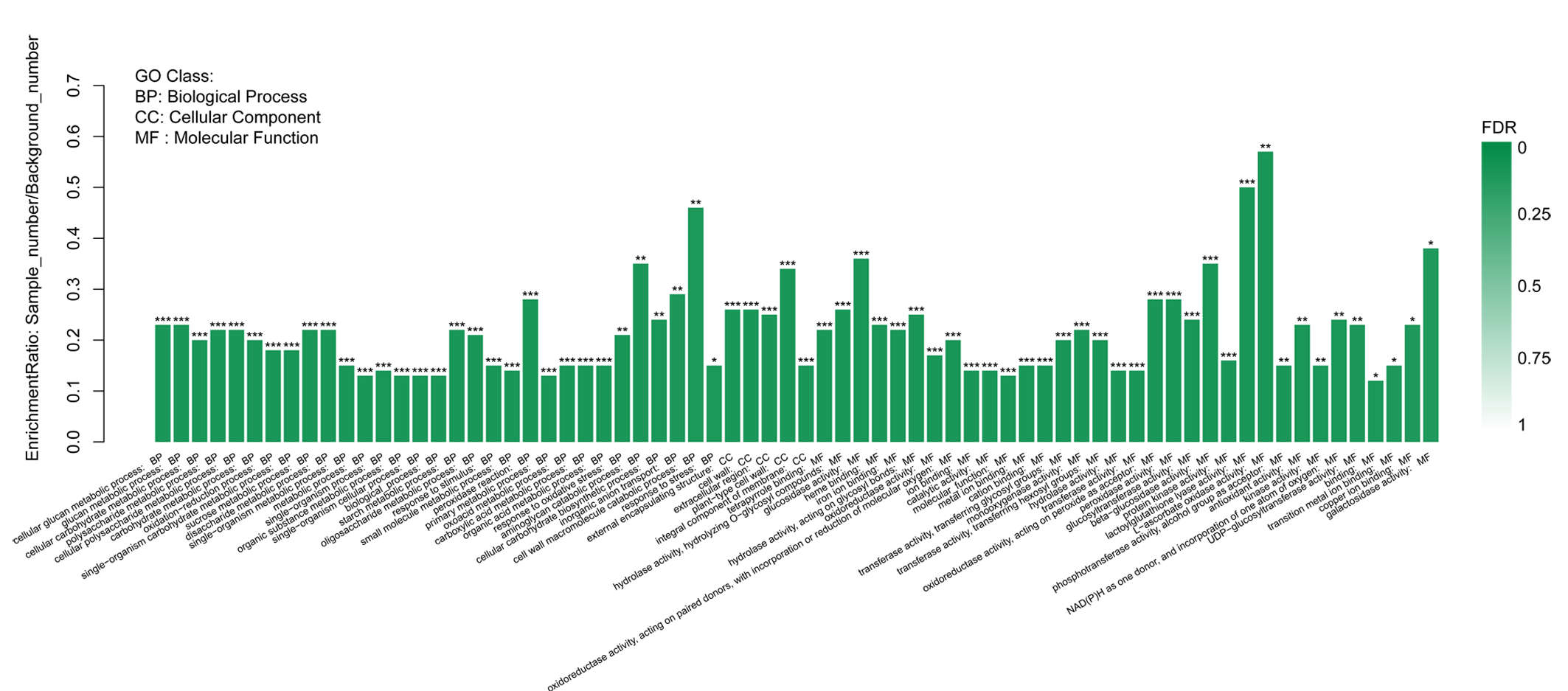

Supplement: Supplementary file 1 [file Image_1.TIF]

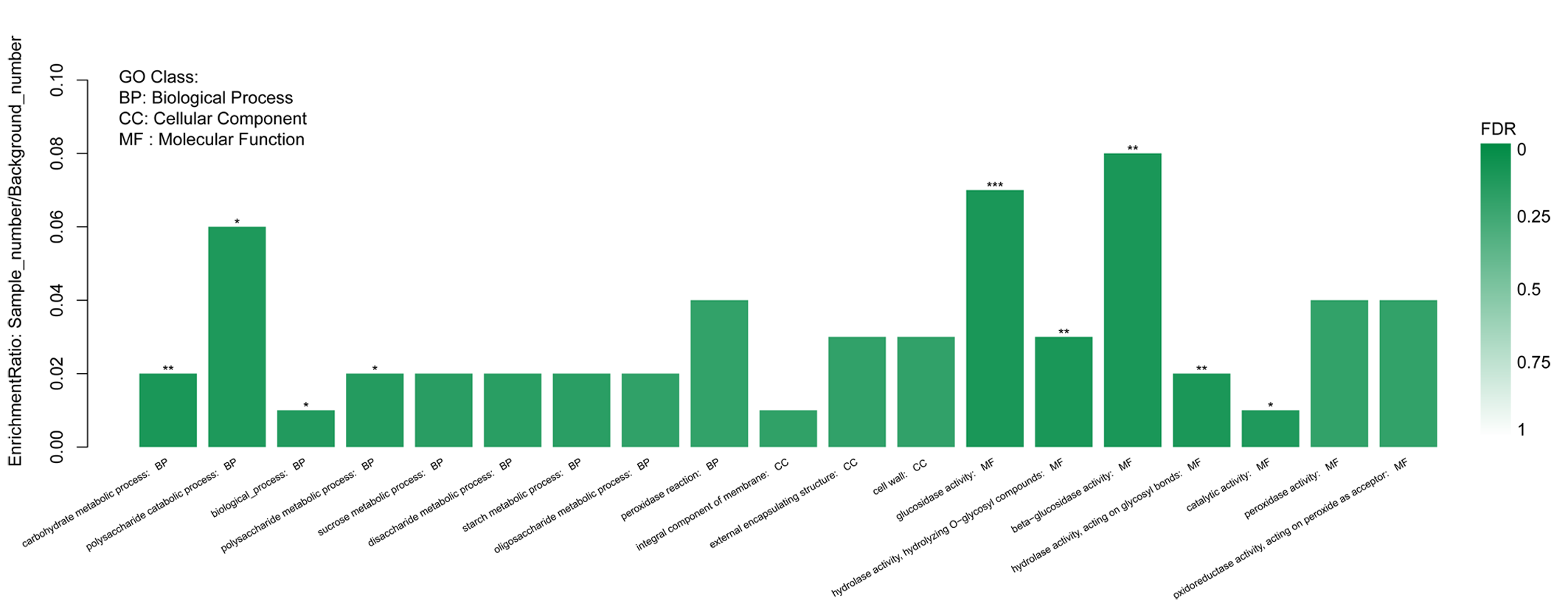

Supplement: Supplementary file 2 [file Image_2.TIF]
